# Comparison of restrictive and liberal transfusion strategies on clinical outcomes in patients with upper gastrointestinal bleeding in the emergency department

**DOI:** 10.1007/s11845-026-04318-x

**Published:** 2026-03-31

**Authors:** Evren Dal, Suna Eraybar

**Affiliations:** University of Health Sciences, Bursa Faculty of Medicine Bursa Sehir Training and Research Hospital emergency Department, Bursa, Nilüfer Turkey

## Introduction

Acute upper gastrointestinal (UGI) bleeding is a common presentation in emergency departments and represents a clinical emergency associated with substantial morbidity and mortality. According to data reported in the literature, the annual incidence ranges between 50 and 200 per 100,000 individuals, with in-hospital mortality rates reported between 10% and 30% [1–3]. The sources of bleeding are generally classified as variceal and non-variceal, with peptic ulcers, esophageal varices, Mallory–Weiss tears, malignancies, and vascular lesions being among the most common etiologies [4–6]. While variceal bleeding typically develops in the setting of portal hypertension, non-variceal bleeding is most often related to ulcerative lesions [7, 8]. Regional differences may influence this distribution [5, 6].

Management of patients with UGI bleeding includes stabilization of hemodynamics, intravenous fluid resuscitation, proton pump inhibitor therapy, endoscopic interventions, and blood transfusion when indicated [7, 9–11]. This frequently encountered clinical condition places a significant burden on both patients and healthcare systems.

Blood transfusion is a complex therapeutic intervention that serves not only to replace blood loss but also to improve oxygen-carrying capacity, support coagulation, and treat hypovolemic shock [12–14]. Transfusion is considered comparable to tissue or organ transplantation and may lead to serious complications if administered outside absolute indications [12, 15]. Therefore, within the framework of patient blood management (PBM), the primary goals include prevention of anemia, preservation of red cell mass, maintenance of hemostasis, and avoidance of unnecessary transfusions [16–18].

The World Health Organization defines anemia as a hemoglobin (Hb) level < 13 g/dL in men, < 12 g/dL in women, and < 11 g/dL in pregnant women [19]. However, in clinically stable patients, a transfusion threshold of 7 g/dL is commonly recommended and supported by multiple clinical guidelines [20–23]. Adverse effects associated with transfusion include hemolytic reactions, alloimmunization, infection risk, immunomodulation, and increased mortality, highlighting the importance of careful decision-making [13–15, 18, 24]. Currently, the use of blood components such as packed red blood cells, fresh frozen plasma, and platelets instead of whole blood aims to improve transfusion safety [18].

In patients with UGI bleeding, transfusion strategies are generally categorized into two main approaches: restrictive and liberal. The restrictive strategy typically recommends transfusion when Hb levels fall below 7 g/dL, whereas the liberal strategy advocates initiating transfusion at Hb levels of 9–10 g/dL [20, 21, 23]. In a multicenter randomized controlled trial conducted by Villanueva et al., the restrictive strategy was reported to be superior to the liberal approach in terms of mortality, rebleeding rates, and complications [25, 26]. Similarly, Cochrane reviews and observational studies have demonstrated that restrictive transfusion strategies are at least as safe as liberal strategies and may even be more advantageous in certain clinical scenarios [23, 24, 26, 27].

Conversely, some evidence suggests that liberal transfusion, particularly in variceal bleeding associated with portal hypertension, may increase portal pressure and consequently elevate the risk of rebleeding [12, 28, 29]. Feasibility studies such as TRIGGER have shown that liberal transfusion strategies do not provide short-term clinical benefits [30]. International guidelines currently recommend an Hb threshold of 7 g/dL for non-variceal UGI bleeding and 8 g/dL for variceal bleeding [7, 8, 31–33].

Unnecessary transfusions not only impose an economic burden but also increase the risk of complications that threaten patient safety [14, 15, 34, 35]. In the emergency department setting, transfusion decisions often need to be made rapidly, making the application of patient-specific and evidence-based transfusion strategies even more critical. Although existing studies support the effectiveness of restrictive transfusion strategies in UGI bleeding, there remains a need for real-world data to further validate these approaches [23–27].

Therefore, this study was designed to compare clinical outcomes in patients grouped according to hemoglobin levels at the time of transfusion (< 7 g/dL and 7–10 g/dL).The findings of this study may contribute to the evaluation of transfusion indications and the development of patient management protocols in emergency care.

## Materials and methods

### Study design and setting

This retrospective, descriptive, and comparative observational study was conducted in the Emergency Department of a tertiary University Hospital between January 1, 2022, and October 30, 2023. Approval for the study was obtained from the local ethics committee (Ethics Committee Decision No:2019 KAEK 1402023-21/8).

### Study population

Adult patients who presented to the emergency department with complaints of hematochezia and/or melena, were diagnosed with upper gastrointestinal (UGI) bleeding, and received blood product transfusion were included in the study. The diagnosis of UGI bleeding was confirmed based on clinical evaluation and/or endoscopic findings.

Patients younger than 18 years of age, those diagnosed with lower gastrointestinal bleeding, patients with missing data, and patients with active bleeding related to malignancy were excluded from the study.

### Data collection

Data were retrospectively obtained from the hospital electronic medical record system. The following variables were recorded: age, sex, known comorbidities, presenting complaints, vital signs at admission, and laboratory parameters, including complete blood count results. Hemoglobin levels before and after transfusion, as well as the type and amount of transfused blood products, were also analyzed.

In addition, patient disposition from the emergency department (discharge, ward admission, or intensive care unit admission), emergency department revisit, and mortality data within a 3-month follow-up period were evaluated.

### Grouping and definitions

Patients were categorized into two groups based on hemoglobin levels at the time of transfusion, rather than predefined institutional transfusion protocols:

- Restrictive transfusion group: patients who received transfusion with a target hemoglobin level of < 7 g/dL at the time of transfusion.
- Liberal transfusion group: patients who received transfusion with a target hemoglobin level of 7–10 g/dL at the time of transfusion.

The two groups were compared in terms of demographic characteristics, clinical findings, post-transfusion hemoglobin levels, need for repeat transfusion, 3-month survival rates, and hospital admission outcomes.

### Statistical analysis

Statistical analyses were performed using SPSS software (version XX, IBM Corp., Armonk, NY, USA). The distribution of continuous variables was assessed using the Shapiro–Wilk test. For non-normally distributed data, the Mann–Whitney U test was used. Categorical variables were analyzed using the Pearson chi-square test or Fisher’s exact test, as appropriate.

Multivariable analyses were conducted using logistic regression models. A p-value < 0.05 was considered statistically significant.

## Results

This retrospective study was conducted on patients evaluated in the emergency department for upper gastrointestinal (UGI) bleeding between January 1, 2022, and October 30, 2023. A total of 1,730 patients were initially assessed during the study period. Patients who did not receive transfusion, those who did not receive packed red blood cell transfusion, and patients with incomplete follow-up data were excluded. The final analysis included 1,349 patients who received transfusion and had complete clinical data.

Among these patients, 148 individuals received transfusion at hemoglobin levels greater than 10 g/dL. As these patients fell outside the conventional definitions of restrictive and liberal transfusion strategies, they were excluded from further comparative analyses. Potential reasons for transfusion at higher hemoglobin levels may include active ongoing bleeding, hemodynamic instability, comorbid conditions, or clinician-based decision-making. After these exclusions, a total of 1,180 patients were included in the final analysis.

**Fig. 1:**
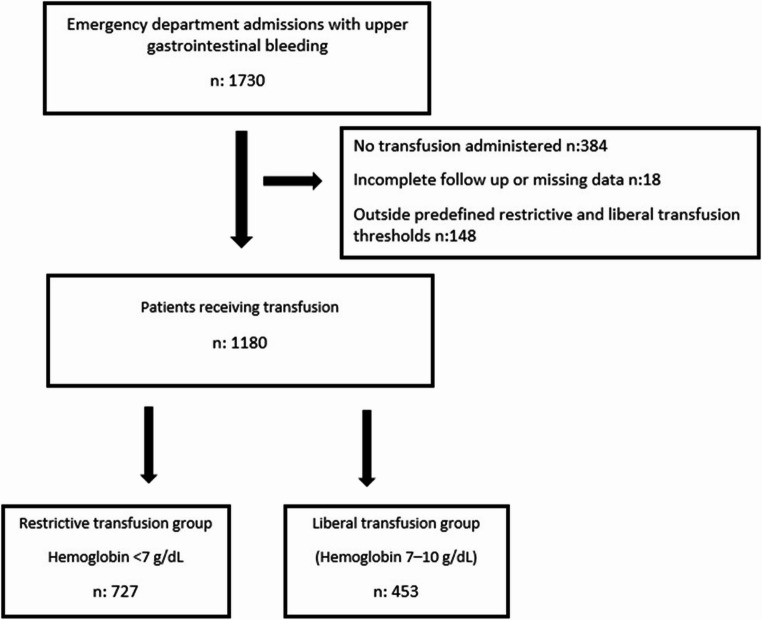
Study Flow Diagram

Patients with hemoglobin levels below 7 g/dL at the time of transfusion (*n* = 727) were assigned to the restrictive transfusion group, while patients with hemoglobin levels between 7 g/dL and 10 g/dL (including 10 g/dL; *n* = 453) comprised the liberal transfusion group. The study flow diagram is summarized in Fig. 1.

The median age of the study population was 69 years (interquartile range [IQR], 58–79 years). Of the 1,180 patients included, 610 (51.7%) were male and 570 (48.3%) were female. Comorbid conditions included diabetes mellitus in 6.7% of patients, hypertension in 12.5%, chronic kidney disease in 8.2%, congestive heart failure in 2.7%, asthma or chronic obstructive pulmonary disease in 3.3%, and a history of malignancy in 9.6%. Baseline demographic and clinical characteristics of the study population are summarized in Table 1.

**Table 1:** Baseline Demographic and Clinical Characteristics of the Study

Age, years, median (IQR)	69 (58–79)
Male, n (%)	610(51.7%)
Female, n (%)	570 (48.3%)
Diabetes mellitus, n (%)	79 (6.7%)
Hypertension, n (%)	148 (12.5%)
Chronic kidney disease, n (%)	97 (8.2%)
Congestive heart failure, n (%)	36 (2.7%)
Asthma / COPD, n (%)	45 (3.3%)
History of malignancy, n (%)	130 (9.6%)

*IQR* interquartile range, *COPD* chronic obstructive pulmonary disease

All patients received packed red blood cell transfusions. In addition, 7.0% of patients received platelet transfusions, and 37.2% received fresh frozen plasma. Combined transfusion of more than one blood component (e.g., packed red blood cells plus fresh frozen plasma or platelets) was administered in 41.9% of cases.

Baseline laboratory parameters included hemoglobin, hematocrit, white blood cell count, renal function markers, and electrolyte levels. Median hemoglobin and hematocrit levels at presentation were 6.50 g/dL (IQR, 5.60–7.60) and 20.60% (IQR, 17.70–23.60), respectively. Median post-transfusion hemoglobin level was 8.40 g/dL (IQR, 7.05–9.40). Laboratory findings and transfusion-related parameters are summarized in Table 2.

**Table 2:** Transfusion Characteristics and Laboratory Findings

Parameter	Median (IQR)
Hemoglobin, g/dL	6.50 (5.60–7.60)
Hematocrit, %	20.60 (17.70–23.60)
White blood cell count (×10⁹/L)	8.17 (5.71–11.88)
Blood urea nitrogen, mg/dL	52.90 (33.80–89.90)
Creatinine, mg/dL	1.07 (0.76–1.64)
eGFR, mL/min/1.73 m²	58.45 (33.90–90.00)
Calcium, mg/dL	8.50 (8.10–8.90)
Sodium, mmol/L	138.00 (135.00–140.00)
Potassium, mmol/L	4.30 (4.00–4.80)
Post-transfusion hemoglobin, g/dL	8.40 (7.05–9.40)
Post-transfusion hematocrit, %	25.30 (22.10–28.30)
Emergency department length of stay, hours	11.00 (4.00–545.50)

*IQR* interquartile range, *WBC* white blood cell count, *BUN* blood urea nitrogen, *eGFR* estimated glomerular filtration rate, *ED* emergency department

When the restrictive and liberal transfusion groups were compared, no statistically significant differences were observed in age distribution (*p* = 0.6428). As expected, hemoglobin and hematocrit levels were significantly higher in the liberal transfusion group compared with the restrictive group (both *p* < 0.001). White blood cell counts did not differ significantly between the groups (*p* = 0.2607). Comparative demographic and laboratory characteristics of the two groups are presented in Table 3.

**Table 3:** Comparison of Demographic and Laboratory Parameters Between Transfusion Groups

Parameter	Restrictive Group Median (IQR)	Liberal Group Median (IQR)	*p* value
Age, years	69.00 (57.00–79.00)	68.00 (59.00–79.00)	0.6428ᵃ
Hemoglobin, g/dL	5.80 (5.10–6.40)	7.90 (7.40–8.70)	< 0.001ᵃ
Hematocrit, %	18.40 (16.20–20.35)	24.30 (22.40–26.20)	< 0.001ᵃ
White blood cell count (×10⁹/L)	8.07 (5.67–11.52)	8.59 (5.86–12.54)	0.2607ᵃ

*IQR* interquartile range, a Mann–Whitney U test

Clinical outcomes, including 28-day mortality, 90-day mortality, intensive care unit (ICU) admission, ward admission, and discharge rates, were compared between the two groups. The 28-day mortality rate was 13.3% in the restrictive transfusion group and 17.4% in the liberal transfusion group; this difference did not reach statistical significance (*p* = 0.066). Similarly, no significant difference was observed in 90-day mortality rates between the groups (*p* = 0.882).

However, discharge rates from the emergency department were significantly higher in the restrictive transfusion group (*p* < 0.001). In contrast, both ward admission and ICU admission rates were significantly higher in the liberal transfusion group (*p* = 0.003 and *p* = 0.002, respectively). Clinical outcome comparisons between the restrictive and liberal transfusion groups are summarized in Table 4.

**Table 4:** Clinical Outcomes According to Transfusion Strategy

Outcome	Restrictive Group (*n* = 727)	Liberal Group (*n* = 453)	*p* value
28-day mortality, n	97	79	0.066ᵇ
90-day mortality, n	50	33	0.882ᵇ
Discharge from ED, n	318	131	< 0.001ᵇ
Ward admission, n	286	219	0.003ᵇ
ICU admission, n	100	95	0.002ᵇ

*ED* emergency department, *ICU* intensive care unit

*b Pearson chi-square test*

## Discussion

This study was conducted to compare the effects of hemoglobin level–based grouping at the time of transfusion (< 7 g/dL vs. 7–10 g/dL) on mortality and clinical outcomes in patients who received blood transfusion due to upper gastrointestinal (UGI) bleeding in the emergency department. Our findings demonstrated that the restrictive transfusion strategy was associated with more favorable discharge and hospitalization outcomes, while no statistically significant difference was observed between the two strategies in terms of mortality rates.

Transfusion thresholds have long been a subject of debate. Although many current guidelines recommend an Hb threshold of < 7 g/dL for transfusion in hemodynamically stable patients, this limit is frequently modified in clinical practice based on patient age, comorbidities, and hemodynamic status. However, in recent years, multiple randomized controlled trials and systematic reviews have shown that restrictive transfusion strategies are at least as safe as liberal strategies and may even be superior in selected patient populations.

In a landmark randomized controlled trial by Villanueva et al., conducted in 921 patients with acute UGI bleeding, the restrictive strategy significantly reduced 45-day mortality (5% vs. 9%), rebleeding rates, and overall complications compared with the liberal approach [26]. Although the difference in mortality did not reach statistical significance in our study (13.3% vs. 17.4%; *p* = 0.066), the observed trend is consistent with the findings reported by Villanueva et al. Furthermore, the similarity in 90-day mortality rates between the two groups (6.9% vs. 7.3%; *p* = 0.882) suggests that neither strategy confers a clear long-term survival advantage.

The TRIGGER trial demonstrated that liberal transfusion did not provide clinical benefit in terms of rebleeding or length of hospital stay, highlighting the potential risks of liberal transfusion strategies, particularly in acute care settings [30]. These findings support the notion that liberal transfusion may expose patients to unnecessary risks without improving clinical outcomes.

On the other hand, some studies have advocated for liberal transfusion strategies in specific clinical scenarios, particularly in patients with underlying cardiovascular disease, where hemoglobin levels below 8–9 g/dL may increase the risk of myocardial ischemia [22]. However, such findings are applicable only to selected patient groups and should not be generalized. Moreover, many of these studies are observational in nature and lack randomization, which limits their methodological robustness [27, 29].

In the present study, ward admission (39.3% vs. 48.3%; *p* = 0.003) and intensive care unit admission rates (13.8% vs. 21.0%; *p* = 0.002) were significantly higher in the liberal transfusion group. These findings suggest that liberal transfusion strategies may be associated with increased healthcare utilization and potentially higher complication rates. Possible explanations include transfusion-related immunomodulation, increased infection risk, and higher likelihood of rebleeding in patients receiving liberal transfusion [13–15].

Consistent with our findings, Cochrane systematic reviews and meta-analyses have reported that restrictive transfusion strategies reduce hospital length of stay and the volume of transfused blood products without increasing mortality or morbidity [23, 24]. This approach aligns closely with patient blood management (PBM) principles, which emphasize not only the correction of anemia but also the minimization of unnecessary transfusions and transfusion-related complications [16, 17].

Overall, although the literature contains some conflicting evidence regarding optimal transfusion thresholds, the prevailing trend supports restrictive transfusion strategies, particularly in patients with UGI bleeding. Prospective and randomized studies in this population have consistently demonstrated that restrictive strategies are associated with lower rebleeding rates and comparable or improved survival outcomes [26].

The primary strength of this study lies in its evaluation of restrictive and liberal transfusion strategies using real-world data from an emergency department setting. However, several limitations should be acknowledged. The retrospective design and lack of standardized transfusion protocols may have introduced heterogeneity in clinical decision-making. Additionally, the absence of detailed endoscopic findings and bleeding etiology (variceal vs. non-variceal) limited the ability to perform subgroup analyses.

## Conclusion

This study comparatively evaluated the clinical effects of restrictive (Hb < 7 g/dL) and liberal (Hb 7–10 g/dL) transfusion strategies in patients who received blood transfusion due to upper gastrointestinal (UGI) bleeding. The findings demonstrated that the restrictive transfusion strategy was associated with higher discharge rates and lower rates of ward and intensive care unit admissions. Although no statistically significant differences were observed in 28-day and 90-day mortality rates, a favorable trend toward clinical safety and effectiveness was observed with the restrictive approach, consistent with existing literature.

These results highlight the importance of avoiding unnecessary transfusions in accordance with patient blood management principles to improve patient safety and optimize healthcare resource utilization. In clinical practice, transfusion decisions should be individualized based not only on hemoglobin levels but also on the patient’s overall clinical status, comorbid conditions, and hemodynamic stability. In this context, restrictive transfusion strategies may represent a more rational and sustainable approach in the management of patients with upper gastrointestinal bleeding.

### Limitations

This study has several limitations inherent to its retrospective and single-center design. First, because data were collected retrospectively from the hospital information system, detailed clinical parameters and endoscopic findings were unavailable for some patients. As a result, important variables such as bleeding etiology (variceal vs. non-variceal) could not be evaluated, precluding subgroup analyses.

Second, transfusion decisions were based on individual clinician judgment rather than standardized protocols, which may have introduced heterogeneity between patient groups. Additionally, although mortality outcomes were assessed, data on rebleeding rates, transfusion-related complications, and specific morbidity outcomes were not available, limiting the assessment of long-term clinical effects.

Furthermore, key factors influencing transfusion decisions—such as hemodynamic parameters, estimated blood loss, and clinical stability—were not systematically recorded, preventing precise adjustment for these confounders. The single-center nature of the study may also limit the generalizability of the findings to other institutions with different patient populations and transfusion practices.

Finally, compared with prospective randomized controlled trials, the observational design of this study limits the ability to establish causal relationships. Therefore, the results should be interpreted cautiously and considered hypothesis-generating, potentially guiding future prospective and randomized investigations.

## Supplementary Information

Below is the link to the electronic supplementary material.

Supplementary Material 1.

Supplementary Material 2.

## References

[1] Lewis JD, Bilker WB, Brensinger C, Farrar JT, Strom BL (2002) Hospitalization and mortality rates from peptic ulcer disease and gastrointestinal bleeding in the 1990s: relationship to sales of nonsteroidal anti-inflammatory drugs and acid suppression medications. Am J Gastroenterol 97:2540–2549. 10.1111/j.1572-0241.2002.06037.x 12385436 10.1111/j.1572-0241.2002.06037.x

[2] Sung JJ, Tsoi KK, Ma TK, Yung MY, Lau JY, Chiu PW (2010) Causes of mortality in patients with peptic ulcer bleeding: a prospective cohort study of 10,428 cases. Am J Gastroenterol 105:84–89. 10.1038/ajg.2009.507 19755976 10.1038/ajg.2009.507

[3] Laine L, Yang H, Chang SC, Datto C (2012) Trends for incidence of hospitalization and death due to gastrointestinal complications in the United States from 2001 to 2009. Am J Gastroenterol 107:1190–1195. 10.1038/ajg.2012.168 22688850 10.1038/ajg.2012.168

[4] Anand CS, Tandon BN, Nundy S (1983) The causes, management and outcome of upper gastrointestinal haemorrhage in an Indian hospital. Br J Surg 70:209–211 6600954 10.1002/bjs.1800700407

[5] Singh SP, Panigrahi MK (2013) Spectrum of upper gastrointestinal hemorrhage in coastal Odisha. Trop Gastroenterol 34:147–150 10.7869/tg.2012.85 23923369

[6] Simon EG, Chacko A, Dutta AK, Joseph AJ, George B (2013) Acute nonvariceal upper gastrointestinal bleeding: experience of a tertiary care center in southern India. Indian J Gastroenterol 32:236–241 23526425 10.1007/s12664-013-0305-6

[7] Barkun AN, Bardou M, Kuipers EJ, Sung J, Hunt RH, Martel M, Sinclair P (2010) International consensus recommendations on the management of patients with nonvariceal upper gastrointestinal bleeding. Ann Intern Med 152:101–113. 10.1059/0003-4819-152-2-201001190-00009 20083829 10.7326/0003-4819-152-2-201001190-00009

[8] de Franchis R (2010) Revising consensus in portal hypertension: report of the Baveno V consensus workshop on methodology of diagnosis and therapy in portal hypertension. J Hepatol 53:762–768. 10.1016/j.jhep.2010.06.004 20638742 10.1016/j.jhep.2010.06.004

[9] Dellinger RP, Levy MM, Carlet JM et al (2008) Surviving Sepsis Campaign: international guidelines for management of severe sepsis and septic shock. Crit Care Med 36:296–327. 10.1097/01.CCM.0000298158.12101.41 18158437 10.1097/01.CCM.0000298158.12101.41

[10] Hébert PC, Wells G, Blajchman MA et al (1999) A multicenter, randomized, controlled clinical trial of transfusion requirements in critical care. N Engl J Med 340:409–417. 10.1056/NEJM199902113400601 9971864 10.1056/NEJM199902113400601

[11] Lacroix J, Hébert PC, Hutchison JS et al (2007) Transfusion strategies for patients in pediatric intensive care units. N Engl J Med 356:1609–1619. 10.1056/NEJMoa066240 17442904 10.1056/NEJMoa066240

[12] Castañeda B (2001) Effects of blood volume restitution following a portal hypertensive-related bleeding in anesthetized cirrhotic rats. Hepatology 33:821–825 11283845 10.1053/jhep.2001.23437

[13] McCormick PA, Jenkins SA, McIntyre N, Burroughs AK (1995) Why portal hypertensive varices bleed and bleed: a hypothesis. Gut 36:1003–1006 10.1136/gut.36.1.100 PMC1382361 7890210

[14] Vengelen-Tyler V (ed) (1996) Noninfectious complications of blood transfusion. Technical Manual, 12th edn. American Association of Blood Banks, Bethesda, pp 558–559

[15] Hillman RS, Ault KA (2002) *Blood component therapy*. In: Hematology in clinical practice, 3rd ed. McGraw-Hill, pp 407–416

[16] Marik PE, Corwin HL (2008) Efficacy of red blood cell transfusion in the critically ill: a systematic review of the literature. Crit Care Med 36:2667–2674 18679112 10.1097/CCM.0b013e3181844677

[17] Beattie WS, Karkouti K, Wijeysundera DN, Tait G (2009) Risk associated with preoperative anemia in noncardiac surgery: a single-center cohort study. Anesthesiology 110:574–581 19212255 10.1097/ALN.0b013e31819878d3

[18] Vincent JL, Jaschinski U, Wittebole X et al (2018) Worldwide audit of blood transfusion practice in critically ill patients. Crit Care 22:102. 10.1186/s13054-018-2018-9 29673409 10.1186/s13054-018-2018-9 PMC5909204

[19] Choorapoikayil S, Zacharowski K, Meybohm P (2016) Patient blood management: is it worth to be employed? Curr Opin Anesthesiol 29:186–191 10.1097/ACO.0000000000000298 26705130

[20] Benoist B, McLean E, Egli I, Cogswell M (eds) (2008) Worldwide prevalence of anaemia 1993–2005: WHO global database on anaemia. World Health Organization, Geneva

[21] Hébert PC, Carson JL (2014) Transfusion threshold of 7 g per deciliter: the new normal. N Engl J Med 371:1459–1461. 10.1056/NEJMe1407693 25270276 10.1056/NEJMe1408976

[22] Likosky DS, Al-Attar PM, Malenka DJ et al (2014) Geographic variability in potentially discretionary red blood cell transfusions after coronary artery bypass graft surgery. J Thorac Cardiovasc Surg 148:3084–3089 25227699 10.1016/j.jtcvs.2014.07.106 PMC5082980

[23] Hearnshaw S, Brunskill S, Doree C, Hyde C, Travis S, Murphy MF (2009) Red cell transfusion for the management of upper gastrointestinal haemorrhage. Cochrane Database Syst Rev 2CD006613. 10.1002/14651858.CD006613.pub2 10.1002/14651858.CD006613.pub2 19370645

[24] Jairath V, Hearnshaw S, Brunskill SJ et al (2010) Red cell transfusion for the management of upper gastrointestinal haemorrhage. Cochrane Database Syst Rev 9:CD006613. 10.1002/14651858.CD006613.pub3 10.1002/14651858.CD006613.pub3 20824851

[25] Hearnshaw SA, Logan RF, Palmer KR, Card TR, Travis SP, Murphy MF (2010) Outcomes following early red blood cell transfusion in acute upper gastrointestinal bleeding. Aliment Pharmacol Ther 32:215–224 10.1111/j.1365-2036.2010.04348.x 20456308 10.1111/j.1365-2036.2010.04348.x

[26] Villanueva C, Colomo A, Bosch A et al (2013) Transfusion strategies for acute upper gastrointestinal bleeding. N Engl J Med 368:11–21. 10.1056/NEJMoa1211801 23281973 10.1056/NEJMoa1211801

[27] Restellini S, Kherad O, Jairath V, Martel M, Barkun AN (2013) Red blood cell transfusion is associated with increased rebleeding in patients with nonvariceal upper gastrointestinal bleeding. Aliment Pharmacol Ther 37:316–322. 10.1111/apt.12170 23205554 10.1111/apt.12170

[28] Blair SD, Janvrin SB, McCollum CN, Greenhalgh RM (1986) Effect of early blood transfusion on gastrointestinal haemorrhage. Br J Surg 73:783–785 3533203 10.1002/bjs.1800731007

[29] Duggan JM (2001) Transfusion in gastrointestinal haemorrhage: if, when and how much? Aliment Pharmacol Ther 15:1109–1113 11472313 10.1046/j.1365-2036.2001.01013.x

[30] Jairath V, Kahan BC, Gray A et al (2015) Restrictive versus liberal blood transfusion for acute upper gastrointestinal bleeding (TRIGGER). Lancet 386:137–144. 10.1016/S0140-6736(14)61999-1 25956718 10.1016/S0140-6736(14)61999-1

[31] Barkun AN, Almadi M, Kuipers EJ et al (2019) Management of nonvariceal upper gastrointestinal bleeding. Ann Intern Med 171:805–822. 10.7326/M19-1795 31634917 10.7326/M19-1795 PMC7233308

[32] de Franchis R (2015) Expanding consensus in portal hypertension. J Hepatol 63:743–752. 10.1016/j.jhep.2015.05.022 26047908 10.1016/j.jhep.2015.05.022

[33] Oakland K, Jairath V, Murphy MF (2018) Gastrointestinal bleeding: transfusion in gastrointestinal bleeding. Transfus Med 28:132–139. 10.1111/tme.12465 28737229 10.1111/tme.12446

[34] Carson JL, Hill S, Carless P, Hébert P, Henry D (2002) Transfusion triggers: a systematic review of the literature. Transfus Med Rev 16:187–199. 10.1053/tmrv.2002.33461 12075558 10.1053/tmrv.2002.33461

[35] Hébert PC, Wells G, Blajchman MA et al (1999) A multicenter, randomized, controlled clinical trial of transfusion requirements in critical care. N Engl J Med 340:409–417 9971864 10.1056/NEJM199902113400601

